# Anti-Endotoxin 9-Meric Peptide with Therapeutic Potential for the Treatment of Endotoxemia

**DOI:** 10.4014/jmb.2011.11011

**Published:** 2020-11-20

**Authors:** Manigandan Krishnan, Joonhyeok Choi, Sungjae Choi, Yangmee Kim

**Affiliations:** Department of Bioscience and Biotechnology, Konkuk University, Seoul 05029, Republic of Korea

**Keywords:** Antimicrobial peptide, LPS, endotoxemia, gram-negative infection, LPS binding assay, STD-NMR

## Abstract

Inflammatory reactions activated by lipopolysaccharide (LPS) of gram-negative bacteria can lead to severe septic shock. With the recent emergence of multidrug-resistant gram-negative bacteria and a lack of efficient ways to treat resulting infections, there is a need to develop novel anti-endotoxin agents. Antimicrobial peptides have been noticed as potential therapeutic molecules for bacterial infection and as candidates for new antibiotic drugs. We previously designed the 9-meric antimicrobial peptide Pro9-3 and it showed high antimicrobial activity against gram-negative bacteria. Here, to further examine its potency as an anti-endotoxin agent, we examined the antiendotoxin activities of Pro9-3 and elucidated its mechanism of action. We performed a dye-leakage experiment and BODIPY-TR cadaverine and limulus amebocyte lysate assays for Pro9-3 as well as its lysine-substituted analogue and their enantiomers. The results confirmed that Pro9-3 targets the bacterial membrane and the arginine residues play key roles in its antimicrobial activity. Pro9-3 showed excellent LPS-neutralizing activity and LPS-binding properties, which were superior to those of other peptides. Saturation transfer difference-nuclear magnetic resonance experiments to explore the interaction between LPS and Pro9-3 revealed that Trp^3^ and Tlr^7^ in Pro9-3 are critical for attracting Pro9-3 to the LPS in the gram-negative bacterial membrane. Moreover, the anti-septic effect of Pro9-3 in vivo was investigated using an LPS-induced endotoxemia mouse model, demonstrating its dual activities: antibacterial activity against gram-negative bacteria and immunosuppressive effect preventing LPS-induced endotoxemia. Collectively, these results confirmed the therapeutic potential of Pro9-3 against infection of gram-negative bacteria.

## Introduction

Antimicrobial peptides (AMPs) exert broad-spectrum antibacterial activities and have therefore emerged as an alternative type of antibiotics with diverse structures and different modes of action [1]. Accordingly, the development of new AMPs holds promise as a novel method for combating multidrug-resistant (MDR) bacteria [2, 3]. Since most new antibiotics have failed in clinical trials against MDR gram-negative bacteria due to the complexity of their bacterial membranes, the development of new peptide antibiotics targeting these membranes has great importance [4, 5]. Lipopolysaccharide (LPS) is a component of the outer membrane of gram-negative bacteria and is released in the host cell upon infection. LPS is recognized by Toll-like receptor (TLR) 4, which is then activated by LPS to induce the production of pro-inflammatory cytokines that activate the host immune response. However, an uncontrolled inflammatory reaction activated by LPS can lead to sepsis, a systemic inflammatory response to bacterial infection [6-9].

Since the discovery of penicillin as the first antibiotic agent, various classes of antibiotics have been developed that are generally effective in treating bacterial infections [10]. For example, imipenem, an effective carbapenem antibiotic, is highly potent against gram-negative bacteria [11, 12]. However, the appearance of carbapenem-resistant gram-negative bacteria represents an emerging threat and thus the development of new antibiotics is necessary [13, 14]. Since AMPs have dual activities—effective antibacterial activities as well as an immunosuppressive effect—they are promising as potential therapeutics. For example, colistin is a potent AMP against gram-negative bacteria; it binds to the released LPS and is now used as a last-resort therapeutic. However, as colistin is associated with severe kidney toxicity, extensive effort has been focused on developing new colistin-derived peptide antibiotics via chemical and sequence modifications [15, 16]. Similarly, LL-37 is another well-known LPS-neutralizing AMP that effectively prevents initiation of the inflammatory response but shows high cytotoxicity to mammalian cells [17].

To overcome these limitations, we previously designed 9-mer peptides based on the sequence of protaetiamycine, an insect defensin, by optimizing the cationicity and hydrophobicity of the peptides [18, 19]. We subsequently confirmed that Pro9-3 (RLWLAIWRR-NH_2_), with two Trp amino acids and its enantiomer Pro9-3D, showed strong antibacterial activities [20]. Pro9-3D could potently inhibit gram-negative bacteria but showed high cytotoxicity to mammalian cells, whereas Pro9-3 showed potent LPS-neutralizing activities, demonstrating its potential for inhibiting inflammatory signaling.

Accordingly, in the present study we investigated the mechanism underlying the anti-endotoxin activity of Pro9-3 using nuclear magnetic resonance spectroscopy and in vitro LPS-binding assays. We also examined its potency as a candidate therapeutic using an in vivo mouse endotoxemia model of LPS-induced septic shock. Our results offer a foundation for strategies to develop potent anti-endotoxin peptides for treatment of endotoxemia due to gram-negative bacteria infection.

## Materials and Methods

### Peptide Synthesis

All Pro9-3 peptide series listed in Table 1 were synthesized by solid-phase synthesis and purified by reversed-phase preparative high-performance liquid chromatography as described previously [21]. Molecular masses of peptides were measured by a matrix-assisted laser-desorption ionization-time-of-flight (MALDI-TOF) mass spectrometer at Ochang Korea Basic Science Institute (KBSI, Korea).

### Bacterial Strains and Antimicrobial Activity

The gram-negative bacterial strains *Escherichia coli* (KCTC 1682) and *Acinetobacter baumannii* (KCCM 40203) were purchased from the Korean Collection for Type Cultures (Korea) and the Korean Culture Center of Microorganisms (Korea), respectively. The MDR gram-negative strains *Escherichia coli* CCARM 1229, *Escherichia coli* CCARM 1238, *Acinetobacter baumannii* CCARM 12010, and *Acinetobacter baumannii* CCARM 12220 were obtained from the Culture Collection of Antibiotic-Resistant Microbes at Seoul Women’s University (South Korea).

The antibacterial properties of all peptides were determined by measuring the Minimum Inhibitory Concentration (MIC) as described previously [22]. In brief, the MIC was defined as the concentration of peptides that inhibited 99% of the bacterial growth. Two-fold serial dilutions of respective peptides were inoculated in 96-well plates in Mueller-Hinton medium, followed by the addition of bacterial suspensions (2 × 10^5^ CFU/ml) at the log growth phase. The plates were incubated at 37°C for 16 h, and absorbance was read at 600 nm using a SpectraMAX microplate reader (Molecular Devices, USA).

### Cytotoxicity Measurements

The capacity of peptides in the lysis of erythrocytes was analyzed using sheep red blood cells (RBCs) as described previously [22]. Cytotoxicity was further assessed in mouse macrophage RAW264.7 cells, which were cultured in Dulbecco’s modified Eagle medium (Thermo Fischer Scientific Inc., USA) enriched with fetal bovine serum (10%) and antibiotics (penicillin/streptomycin, 100 IU/ 100 mg/L) at 37°C in a CO_2_ incubator. The cytotoxic effect of Pro9-3 series peptides against RAW264.7 cells was then determined using WST-8 Cell Proliferation Assay Kit (Biomax Co. Ltd., Korea). The analysis was carried out according to our previously reported method [19].

### Calcein Dye Leakage Assay

Large unilamellar vesicles (LUVs) composed of egg yolk phosphatidylethanolamine (EYPE)/egg yolk phosphatidylglycerol (EYPG) (7:3, w/w) entrapped with calcein dye was used to determine the bacterial membrane permeability of peptides [23]. The membrane permeability by peptides was observed by quantifying the fluorescence (excitation/emission λ = 490/520 nm) of calcein leakage from the EYPE/EYPG LUVs using an RF-5301PC spectrophotometer (Shimadzu, Japan).

### Limulus Amebocyte Lysate (LAL) Assay

To check the endotoxin clearance properties of peptides, LAL assay was performed according to the protocol provided in the kit (Pierce LAL Chromogenic Endotoxin Quantitation Kit, Thermo Fisher Scientific). Escalating concentrations of peptides (up to 50 μM) were added with 2 ng/ml LPS (*E. coli* O111:B4, Sigma-Aldrich, USA) for 15 min at 37°C. To this content, an equal volume of LAL reagent was added and incubated for 15 min and then chromogenic substrate was added. The contents were incubated for another 10 min and 25% acetic acid was used to stop the reaction. The end color (yellow) formed due to reduction of substrate was read at 405 nm. LL-37 was used as a control.

### Peptide-LPS Binding Assay

The capacity of Pro9-3 series peptides to bind with LPS was analyzed using a fluorescent probe, BODIPY-TR cadaverine (BC) (Thermo Fisher Scientific), as described previously [24]. Initially, the probe complex was prepared by incubating LPS (50 mg/ml) with 5 mg/ml BC in a 50 mM Tris buffer (pH 7.4) for 6 h at room temperature. Varying concentrations of peptides (1–128 μM) were added to a 96-well, dark fluorescence plate and allowed to interact with the LPS–BC complex for 30 min. The fluorescence intensity was recorded at an excitation wavelength of 580 nm and emission wavelength of 620 nm using a fluorescence microplate reader (Molecular Devices).

### Saturation Transfer Difference (STD) NMR Experiments

Pro9-3 (0.5 mM) was added to 15 mM LPS in 99.9% D_2_O at pH 5.9. LPS was prepared as described previously [22]. STD-NMR experiments were then performed to assess the peptide-LPS interaction using a Bruker Avance 900 spectrometer (Bruker Corporation, USA) at Korea Basic Science Institute (Korea). The STD-NMR experiments were performed with saturation of LPS resonances at -2.0 ppm selectively and 40 ppm for reference spectra, 45-ms Gaussian-shaped pulses with a 100-ms delay between each pulse, and a total saturation time of 2 s. Subtraction of the on-resonance from the off-resonance spectra provided the difference spectrum.

### Measurement of Antiseptic Activity of Peptides in LPS-Induced Endotoxemia Mouse Model

Four-week-old female ICR (Institute of Cancer Research) mice (24-25g) were procured from Orient Bio (Korea). The animals were acclimatized for one week under ambient temperature (25 ± 2°C) and pyrogen-free conditions. The experiment was performed according to the Institutional Animal Care and Use Committee (IACUC) of Konkuk University, South Korea (IACUC No. KU19197-3).

Animals were randomly sorted into four groups (five mice/group). The control group was intraperitoneally (i.p.) injected with PBS while the peptide control group received only Pro9-3 (1 mg/kg), the LPS group received only LPS (15 mg/kg, i.p.), and the treatment group (Pro9-3 + LPS) received i.p. injection of Pro9-3 (1 mg/kg) for 1 h followed by LPS stimulation for 16 h. The blood and lung tissues were collected and processed for analysis as described previously [25, 26]. Briefly, serum endotoxin levels were quantified according to the kit protocol using a Pierce LAL Chromogenic Endotoxin Quantitation Kit (Thermo Fisher Scientific). The liver (AST and ALT), and kidney (BUN) markers in serum and the inflammatory cytokine release in the serum and lung lysates were quantified as described previously [25, 26].

### Statistical Analysis

All statistical analyses were performed using the GraphPad Prism software 8.0 for windows (GraphPad Software, USA). The experiments were performed in triplicate and the values are expressed as mean ± SEM. Non-linear regression curve fit was employed for RBC lysis, calcein dye leakage and BC-displacement analysis. Statistical significance (*p* < 0.05) was determined using one-way ANOVA with Tukey's test.

## Results

### Properties of Peptides

Physicochemical properties of peptides such as hydrophobicity, cationicity, helicity and amphipathicity are critical factors in the antibacterial activity of peptides and their interactions with bacterial membranes [27]. Net charge is the prime factor for effective electrostatic interaction with negatively charged bacterial membranes [28]. In our previous study, Pro9-3D, an enantiomer of Pro9-3, showed more potent antibacterial activities compared to Pro9-3 [20]; however, Pro9-3D also showed more severe cytotoxicity against mammalian cells. Therefore, we further designed the peptides Pro9-3-K and Pro9-3D-K based on Pro9-3 and Pro9-3D sequences to examine the role of arginine (Arg) residue on antibacterial activity and cytotoxicity by substituting Arg with lysine (Lys). As shown in Table 1, Pro9-3 showed amphipathic properties, and substitution of Arg with Lys did not influence these properties. Pro9-3 has hydrophobic residues “LWLAIW” sequentially, which could potentially increase their antibacterial activities.

### Antibacterial Activities of Peptides

The antimicrobial activities of Pro9-3, Pro9-3D, Pro9-3-K and Pro9-3D-K against gram-negative bacteria and MDR gram-negative bacteria were tested to determine their potency (Table 2). Pro9-3 and Pro9-3D showed much higher antibacterial activity than Pro9-3K and Pro9-3D-K, suggesting that Lys substitution in Pro9-3 for Arg lowered the antibacterial activity. Arg and Lys are two positively charged basic amino acids and they play important roles in stabilizing protein or peptide structures by forming electrostatic interactions. However, the guanidinium group of Arg allows stronger interactions with negatively charged bacterial membrane, resulting in higher antibacterial activity of Pro9-3 compared to that of Pro9-3K. The geometric means (GM) of MICs were assessed to evaluate the antibacterial activities of Pro9-3 series against tested bacterial strains. Only Pro9-3 and Pro9-3D inhibited bacterial growth effectively over a range of MICs close to those of melittin, which was the positive control peptide (GM = 14.4 μM). These results indicated that both Pro9-3 and Pro9-3D are effective against all tested gram-negative bacteria.

### Cytotoxicity of Peptides

**Fig. 1:**
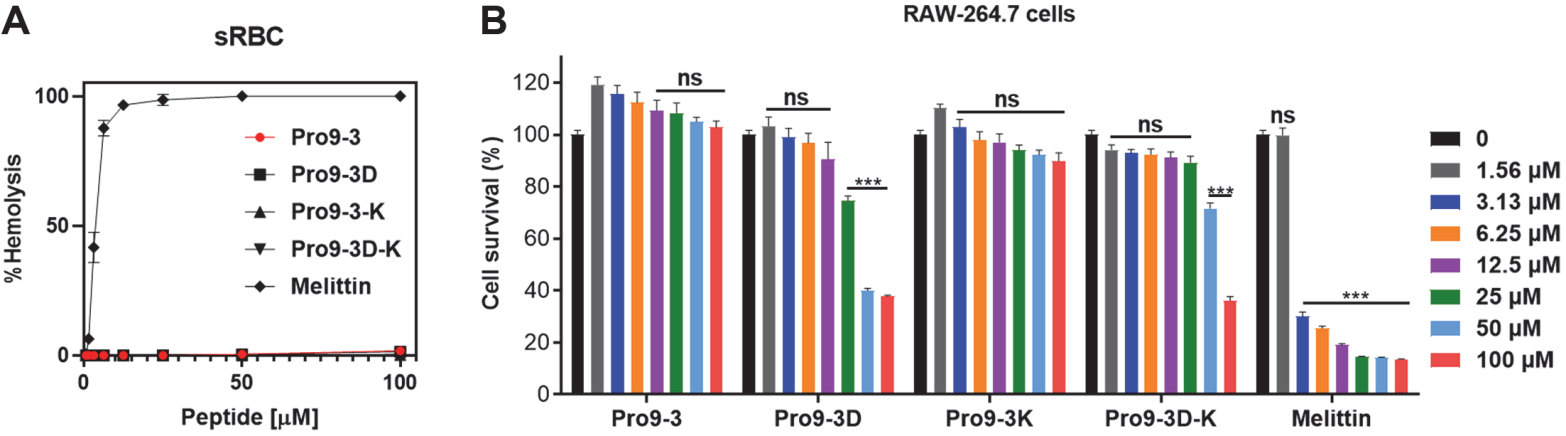
Cytotoxicities of peptides. (**A**) Dose-dependent hemolytic activities in sheep red blood cell (sRBC) caused by peptides. (**B**) Dose-response cytotoxicities of the Pro9-3 and its analogs against RAW264.7 cells for 24 h. Melittin was used as a positive control. The values are expressed as the mean ± SEM of three independent experiments: *p < 0.05; **p < 0.01; ***p < 0.001; ns, not significant.

We next determined the hemolytic and cytotoxic activities of Pro9-3 series peptides. As shown in Fig. 1A, incubation of sRBCs with an escalated dose of all peptides did not induce hemolysis while melittin showed severe cytotoxicity even at low concentration. As indicated in Table 2, Pro9-3D showed good relative selective index (31.25), followed by Pro9-3 (10); the values of both were substantially higher than those of Pro9-3-K (3.125) and Pro9-3D-K (6.94). Furthermore, the in vitro cytotoxicity (Fig. 1B) of peptides against RAW264.7 macrophage cells revealed that Pro9-3 and Pro9-3K resulted in a 100% survival rate, whereas the control peptide melittin showed only 13.7% survival of the macrophages when provided at a concentration of 100 μM. By contrast, Pro9-3D and Pro9-3D-K exhibited significant toxic effects with 40% and 71% survival, respectively, even at 50 μM. Based on the above findings, Pro9-3 showed notable antibacterial activity and it is non-toxic to mammalian cells while Pro9-3D cannot be a peptide antibiotic candidate due to its cytotoxicity against mammalian cells.

### Mechanism of Antibacterial Activities Against Gram-Negative Bacteria

**Fig. 2:**
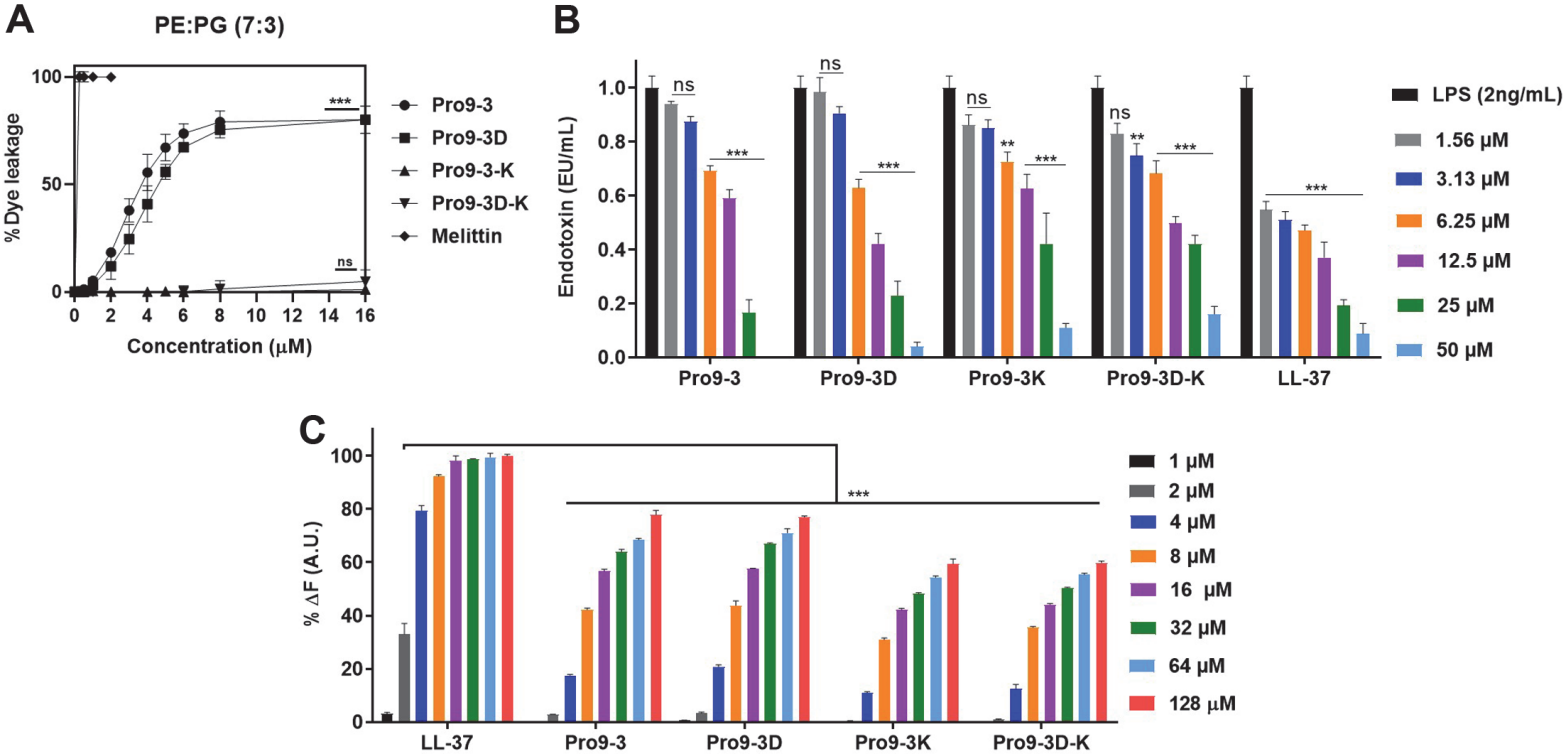
Efficacy of peptides in bacterial membrane permeability and interaction with lipopolysaccharide (LPS). (**A**) Concentration-dependent calcein leakage induced by peptides against egg yolk L-α-phosphatidylethanolamine (EYPE)/egg yolk L-α-phosphatidylglycerol (EYPG) (7:3, w/w) large unilamellar vesicles (LUVs). (**B**) The endotoxin-neutralizing ability of the peptides and LL-37 (positive control): the degree of inhibition, expressed in endotoxin units (EU)/ml signifies the LPSneutralizing properties of the peptides. (**C**) The relative binding affinity of Pro9-3 and its analogs to LPS measured by the change in fluorescence. Dose-dependent BODIPY-TR-cadaverine displacement from *E. coli* (O111:B4) LPS induced by treatment with the peptides. All data represent the mean ± SEM of three independent experiments. Data were analyzed by oneway analysis of variance and Tukey’s post-hoc test: *p < 0.05; **p < 0.01; ***p < 0.001; ns, not significant.

Fig. 2A shows the dose-response relationship of the peptide-induced calcein release based on the percentage of calcein leakages 3 min after peptide exposure to EYPE/EYPG LUVs. Pro9-3 and Pro9-3D peptides caused more than 60% leakage at 6 μM and more than 80% leakage at 16 μM whereas Pro9-3-K and Pro9-3D-K caused only 1.2% and 4.9% leakage even at a high concentration of 16 μM. These results agreed well with the respective antibacterial activities of the peptides, demonstrating the key role of the Arg residue in effective electrostatic interaction with the bacterial membrane, which results in fatal damage to the membrane.

### Pro9-3 Interacts Effectively with LPS

To evaluate the peptide-LPS interaction, LAL assay was performed (Fig. 2B). The results suggested that all peptides significantly neutralized LPS in a dose-dependent manner (Fig. 2A). At 50 μM, Pro9-3, Pro9-3D, Pro9-3-K and Pro9-3D-K effectively neutralized LPS by 100%, 95.9%, 88.9%, and 83.9%, respectively, which showed a comparable or superior effect to the well-known LPS-neutralizing agent, LL-37, with 91% neutralizing activity. Pro9-3 showed the best neutralizing capacity among all peptides.

The BC probe-based displacement assay (Fig. 2C) further showed that LPS has BC quenching ability upon interaction; however, when LPS interacted with the peptides, the BC displacement was alleviated. Incubation of all peptides (1 μM to 128 μM) in LPS-BC complex showed strong dose-dependent enhancement in BC fluorescence when compared to the LPS-neutralizing peptide LL-37. At 128 μM, Pro9-3, Pro9-3D, Pro9-3-K and Pro9-3D-K increased the BC displacement to 76.3%, 76.8%, 61.3%, 60.5%, respectively. Distinctively, Pro9-3 and Pro9-3D showed greater LPS-binding capacity than Pro9-3-K and Pro9-3D-K. Collectively, these results further suggested that Arg is responsible for the effective electrostatic interaction between the peptide and LPS. Therefore, Pro9-3 can be a potent anti-endotoxin peptide without cytotoxicity and therefore we further examined its mechanism of action and potency in vivo.

### Interaction Between LPS and Pro9-3 Determined by STD-NMR

**Fig. 3:**
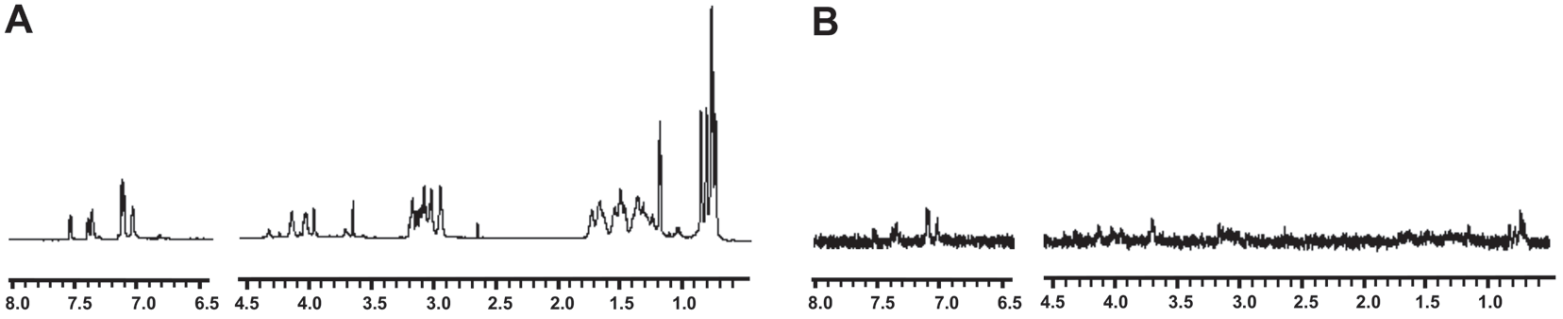
Saturation transfer difference (STD)-nuclear magnetic resonance (NMR) spectra representing the interaction between Pro9-3 (0.5 mM) and LPS (0.015 mM) in the aromatic ring region and aliphatic region. (**A**) ^1^H NMR spectra of Pro9-3 and (**B**) the STD effects. Differences spectrum is established from the resonances belonging to the protons of bound peptide to LPS. In the region from 6.5 to 8.0 ppm, STD effects arising from the aromatic ring protons of Pro9-3 are shown and those from the aliphatic side-chain proton are shown in the region from 0 ppm to 4.5 ppm.

Since Pro9-3 showed the most potent anti-endotoxic activity among all peptides, we further investigated its mechanism of interaction with LPS using STD-NMR to verify the residues nearby the LPS aggregates. ^1^H NMR spectrum of free Pro9-3 in D_2_O is shown in Fig. 3A while STD spectrum of Pro9-3 bound to LPS is shown in Fig. 3B. A difference in the intensity of the peaks between the free Pro9-3 and the LPS-bound Pro9-3 resulted from the saturation transfer between LPS and Pro9-3. Therefore, the STD spectra signals arising from the saturation transfer provided information related to the resonances belonging to the peptide when bound to LPS. The strong STD effect was effectively observed in the region of 7.0-7.6 ppm for the aromatic ring protons of Trp^3^ and Trp^7^, implying that they are in close contact with LPS. In contrast, in the aliphatic region, only weak STD effects were observed. These results implied that Trp^3^ and Trp^7^ in Pro9-3 play critical roles in the hydrophobic interaction with LPS on the gram-negative bacterial membrane.

### Anti-Sepsis Effect of Pro9-3 in LPS-Induced Endotoxemia Mouse Model

**Fig. 4:**
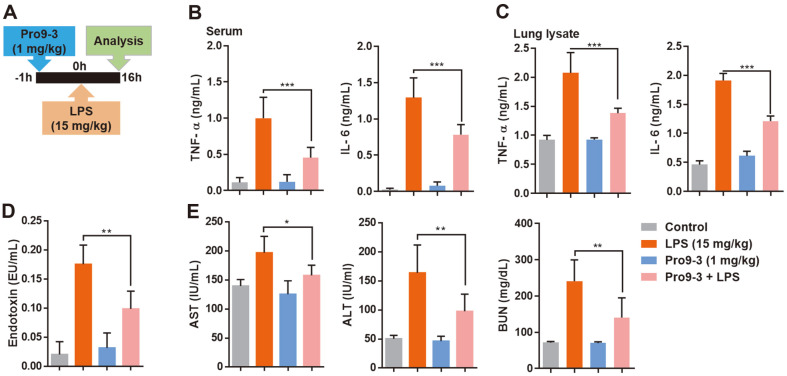
Effects of Pro9-3 on a lipopolysaccharide (LPS)-induced endotoxemia mouse model of septic shock. (**A**) Experimental regimen of endotoxemia model (n = 5). Mice were pre-treated with 1 mg/kg of Pro9-3 and then stimulated with LPS (15 mg/kg, intraperitoneal injection) for 16 h. (**B**) Pro9-3 alleviates the production of inflammatory cytokines (TNF- α and IL-6) in the serum. (**C**) Inhibition of cytokine production in the lung tissue lysates of LPS-challenged mice. (**D**) Pro9-3 normalizes the circulating serum endotoxins in LPS-treated mice. (**E**) Pro9-3 treatment results in effective recovery of aminotransferase (AST), alanine aminotransferase (ALT), and blood urea nitrogen (BUN) levels in the mouse model of endotoxemia. Values are representative of five mice per group (Mean ± SEM) and are statistically significant at *p < 0.05; **p < 0.01; ***p < 0.001; ns, not significant (Tukey’s post-hoc test).

We investigated the therapeutic potential of Pro9-3 using an endotoxemia mouse model stimulated by LPS. Mice challenged with LPS showed aggravated levels of the pro-inflammatory cytokines interleukin (IL)-6 as well as tumor necrosis factor (TNF)-α (Fig. 4). Only the Pro9-3 treatment group showed serum levels of cytokines similar to those of the control group. Specifically, Pro9-3 effectively reduced the serum levels of the inflammatory cytokines TNF-α and IL-6 by 54.5% and 39.4%, respectively, in the LPS-induced endotoxemia mouse model (Fig. 4B). Similarly, Pro9-3 inhibited the production of TNF-α and IL-6 by 33.4% and 36.5%, respectively, in the lung (Fig. 4C).

We then analyzed whether Pro9-3 can remove LPS endotoxin in vivo by using an LAL assay to measure the amount of endotoxin in the serum (Fig. 4D). Pro9-3 reduced the level of endotoxin by 43.5% when injected in an endotoxemia mouse model. Furthermore, the levels of ALT, AST, and BUN were increased by LPS but Pro9-3 treatment reduces them effectively to 19.7%, 59.6%, and 41.7%, respectively (Fig. 4E), demonstrating a protective effect against organ damage in septic shock.

## Discussion

Bacterial infection and associated endotoxins such as LPS from gram-negative bacteria induce severe inflammatory reactions, which can result in sepsis as the most lethal form of response [29, 30]. In particular, the increased emergence of drug-resistant bacterial strains and lack of effective antibiotics has further increased the burden in clinical care units [31]. To overcome this, alternative drugs that can effectively control both infections and inflammatory responses are highly essential. AMPs have been highlighted as alternatives to conventional antibiotics owing to their broad-spectrum bactericidal and immunomodulatory activities against MDR strains, and as promising agents for treating sepsis [15, 23, 32, 33].

Some AMPs such as hLF1-11, P-113, and LL-37 effectively target pathogens through degradation of their outer cell membranes and they show particularly potent ability to neutralize endotoxins while simultaneously killing bacteria [34-37]. Despite these advantageous properties, poor cell selectivity and high cytotoxicity toward mammalian cells are major problems limiting the application of currently available AMPs in disease management. For example, a topical agent derived from magainin (MSI-78) failed in clinical trials since it was not superior to other treatment strategies [38]. The AMP colistin showed potent antimicrobial activities against gram-negative bacteria as well as LPS-binding ability but substantial kidney toxicity limited its use [39, 40]. The cytotoxicity and peptide stability can be improved by chemical and amino acid modifications for the development of new peptide antibiotics [41]. Determining the structure-activity relationships of peptides can provide important data for the design of potent peptides by enhancing or selecting hydrophobic and amphipathic properties that play key roles in antimicrobial activities [42].

Considering these features, we previously developed a short 9-meric antimicrobial peptide termed ‘Pro9-3’ which exerted strong antibacterial activity against gram-negative bacteria [19]. Here, we examined the antibacterial activities and cytotoxicity of Pro9-3, its Lys-substituted analog, and their enantiomers. The results confirmed that cationicity due to the Arg residues in Pro9-3 provides an effective electrostatic interaction with the membrane to cause critical damage to the bacteria, resulting in high antibacterial activity. Since Pro9-3 can neutralize LPS and is a potent anti-endotoxic peptide, we then examined its potential as a therapeutic molecule to treat sepsis in vivo using an endotoxemia mouse model. The specific molecular mechanism by which Pro9-3 targets LPS-induced septic shock remained to be elucidated. There is substantial evidence indicating that many AMPs can induce an anti-inflammatory response through a dual mode of action. First, direct binding of cationic AMPs to LPS via electrostatic interactions was shown to neutralize the anionic amphiphilic lipid A [43]. Second, AMPs were found to bind to the macrophage CD14 receptor and inhibit competitively the interaction of the LPS-LPB complex, thus preventing access to the TLR4 receptor [44]. In this study, we confirmed the binding ability of Pro9-3 to LPS using a fluorescent probe-BC, indicating that Pro9-3 effectively binds to LPS. STD-NMR experiments further revealed that Trp residues in Pro9-3 are important for its hydrophobic interaction with LPS. LPS endotoxin released from the membrane of gram-negative bacteria activates TLR4 and in turn induces production of pro-inflammatory cytokines, which are important in activating the host immune response [6, 45]. Because serious inflammatory responses can induce sepsis, there has been extensive interest in developing peptide antibiotics that simultaneously suppress inflammatory responses and inhibit bacterial growth. Hence, the excellent anti-endotoxin activity of AMPs depends solely on their physicochemical properties. Polymyxin B and LL-37 are well-known LPS-neutralizing AMPs. The presence of circulating LPS can stimulate the excessive production of inflammatory cytokines which causes septic shock. In the case of infection caused by gram-negative bacteria, the mediators for the innate immune system response, especially TLR4, are already significantly enhanced; thus, an anti-endotoxin agent should be able to block the LPS-induced septic shock. Since Pro9-3 showed potency as an anti-endotoxin agent, it can be utilized to treat gram-negative sepsis.

A wealth of research has also demonstrated the applications of various resin-based ligands such as cholestyramine [46] and Sepharose 4B [47] where polymyxin B was covalently attached to selectively adsorb endotoxin from blood [48]. Currently, polymyxin B-immobilized Toraymyxin^®^ cartridge is being effectively used to adsorb endotoxin in septic patients [49]. Pro9-3 has also shown significant LPS-neutralizing capacity, which is an essential attribute for application as a functional biomaterial for endotoxin adsorption and removal of endotoxin from blood.

In summary, we showed that short 9-meric peptide Pro9-3 exhibited these desired dual abilities of LPS neutralizing in an endotoxemia mouse model and strong antibacterial activities against gram-negative bacteria. Taken together, our findings demonstrated that Pro9-3 can be a potent anti-septic peptide and therapeutic agent for treating gram-negative sepsis.

## Figures and Tables

**Table 1:** Physicochemical properties of Pro9-3 series peptides.

Peptides	Sequence^a^	Molecular mass measured	Charge	Hydrophobicity <H>^b^
Pro9-3	RLWLAIWRR-NH2	1269.10	+3	0.692
Pro9-3D	rlwlaiwrr-NH2	1268.27	+3	0.692
Pro9-3-K	KLWLAIWKK-NH2	1184.04	+3	0.687
Pro9-3D-K	klwlaiwkk-NH2	1184.01	+3	0.687

^a^Small letter in sequence represents D-amino acids.

^b^Hydrophobicity<H> was calculated by http://heliquest.ipmc.cnrs.fr/cgi-bin/ComputParams.py

**Table 2:** Antimicrobial activities of the Pro9 series peptides against gram-negative bacterial strains and multidrug-resistant (MDR) bacterial strains.

Microorganisms	Minimal inhibitory concentration (MIC) in μM				

	Pro9-3	Pro9-3D	Pro9-3-K	Pro9-3D-K	Melittin
Gram-negative bacteria					
*E. coli*	16	8	64	16	8
*A. baumannii*	16	4	64	32	8
MDR Gram-negative bacteria					
MDREC 1229	16	8	64	32	16
MDREC 1238	32	4	64	16	32
MDRAB 12010	16	8	64	32	8
MDRAB 12220	16	8	64	32	8

^a^GM	20	6.4	64	28.8	14.4
^b^HC_10_	200	200	200	200	0.8
^c^Relative selective index	10	31.25	3.125	6.94	0.06

^a^The geometric means (GM) are the average values of minimum inhibitory concentration (MIC) values of all tested bacterial strains.

^b^HC_10_ is the peptide concentration which induces 10% hemolysis of sheep red blood cells in vitro.

^c^When no detectable hemolysis was observed at 100 μM, a value of 200 μM was used to calculate the relative selective index. The relative selective index was calculated using HC_10_/GM of the MIC (μM). The peptides with larger values indicate greater cell selectivity.
